# Inhibition of autophagy increases susceptibility of glioblastoma stem cells to temozolomide by igniting ferroptosis

**DOI:** 10.1038/s41419-018-0864-7

**Published:** 2018-08-06

**Authors:** Mariachiara Buccarelli, Matteo Marconi, Simone Pacioni, Ivana De Pascalis, Quintino Giorgio D’Alessandris, Maurizio Martini, Barbara Ascione, Walter Malorni, Luigi Maria Larocca, Roberto Pallini, Lucia Ricci-Vitiani, Paola Matarrese

**Affiliations:** 1 Department of Oncology and Molecular Medicine, Istituto Superiore di Sanità, Rome, Italy; 2 Center for Gender-Specific Medicine, Oncology Unit, Istituto Superiore di Sanità, Rome, Italy; 3 Institute of Neurosurgery, Fondazione Policlinico Universitario “A. Gemelli”, Rome, Italy; 4 Institute of Pathology, Fondazione Policlinico Universitario “A. Gemelli”, Rome, Italy

## Abstract

The role of autophagy in cancer onset and progression appears still controversial. On one hand, autophagy allows cancer cell to survive in unfavorable environmental conditions, on the other hand, once internal energy resources are exhausted, it leads to cell death. In addition, autophagy interpheres with cell cycle progression, de facto exerting a cytostatic activity. Hence, it represents an important target for anticancer therapy. For example, temozolomide (TMZ), of use for glioblastoma (GBM) treatment, appears as capable of inducing autophagy partially inhibiting cancer cell proliferation. However, GBM, a very aggressive brain tumor with poor prognosis even after surgery and radio-chemotherapy, invariably recurs and leads to patient death. Since cancer stem cells have been hypothesized to play a role in refractory/relapsing cancers, in the present work we investigated if autophagy could represent a constitutive cytoprotection mechanism for glioblastoma stem-like cells (GSCs) and if the modulation of autophagic process could affect GBM growth and survival. Thus, in the present study we first evaluated the relevance of autophagy in GBM tumor specimens, then its occurrence in GSCs and, finally, if modulation of autophagy could influence GSC response to TMZ. Our results suggested that, in vitro, the impairing autophagic process with quinacrine, a compound able to cross the blood-brain barrier, increased GSC susceptibility to TMZ. Death of GSCs was apparently due to the iron dependent form of programmed cell death characterized by the accumulation of lipid peroxides called ferroptosis. These results underscore the relevance of the modulation of autophagy in the GSC survival and death and suggest that triggering of ferroptosis in GSCs could represent a novel and important target for the management of glioblastoma.

## Introduction

Glioblastoma (GBM) affects patients of any age, and represents one of the leading cause of cancer-related deaths in the adult population, with median survival being on average little over a year^1,2^. The standard of care for the treatment of GBM consists in maximal resection followed by radiotherapy and concomitant chemotherapy with the alkylating agent temozolomide (TMZ)^3^. However, the majority of GBM cancers progress within 2 years. Within established tumors, a subpopulation of cancer cells with stem cell properties (GBM stem-like cells, GSCs) has been proposed to underlie resistance to therapy and contribute to disease progression^4–6^.

Autophagy is a regulated mechanism of the cell that leads to the disassembly of unnecessary or dysfunctional components. A specific set of genes, called ATGs, is involved in the regulation of autophagy. Among them, the Atg8 family member LC3 appeared as required for autophagosomal membrane closure and for the selective recognition of autophagy substrates. Adaptor proteins, such as the sequestosome 1/p62-like receptors, which directly bind to cargos, contribute to specific molecular targeting. Hence, thanks to this complex mechanism, autophagy can provide energy supply to the cell and can represent a key cytoprotection mechanism allowing cell survival in unfavorable microenvironmental conditions such as those often found by cancer cells^7^. Autophagy may represent a mechanism of resistance to oxidative stress induced by chemotherapeutic drugs and may potentiate cancer cell survival to hypoxia and nutrient starvation due to the frequently defective tumor vascularization. As concerns glioma, autophagy induction has been implicated in the response to TMZ, radiotherapy as well as to molecularly targeted therapies^8–14^. In particular, its inhibition by chloroquine has been suggested to increase overall survival (OS) and the efficacy of conventional treatment with TMZ in retrospective and randomized studies^15–17^. Aim of the present work was to investigate in vitro and in vivo the possible involvement of autophagy, and its modulation in the control of GSC survival and death.

## Results

### Ex vivo analysis of autophagic markers in GBM samples and correlation with patients’ overall survival

**Fig. 1:**
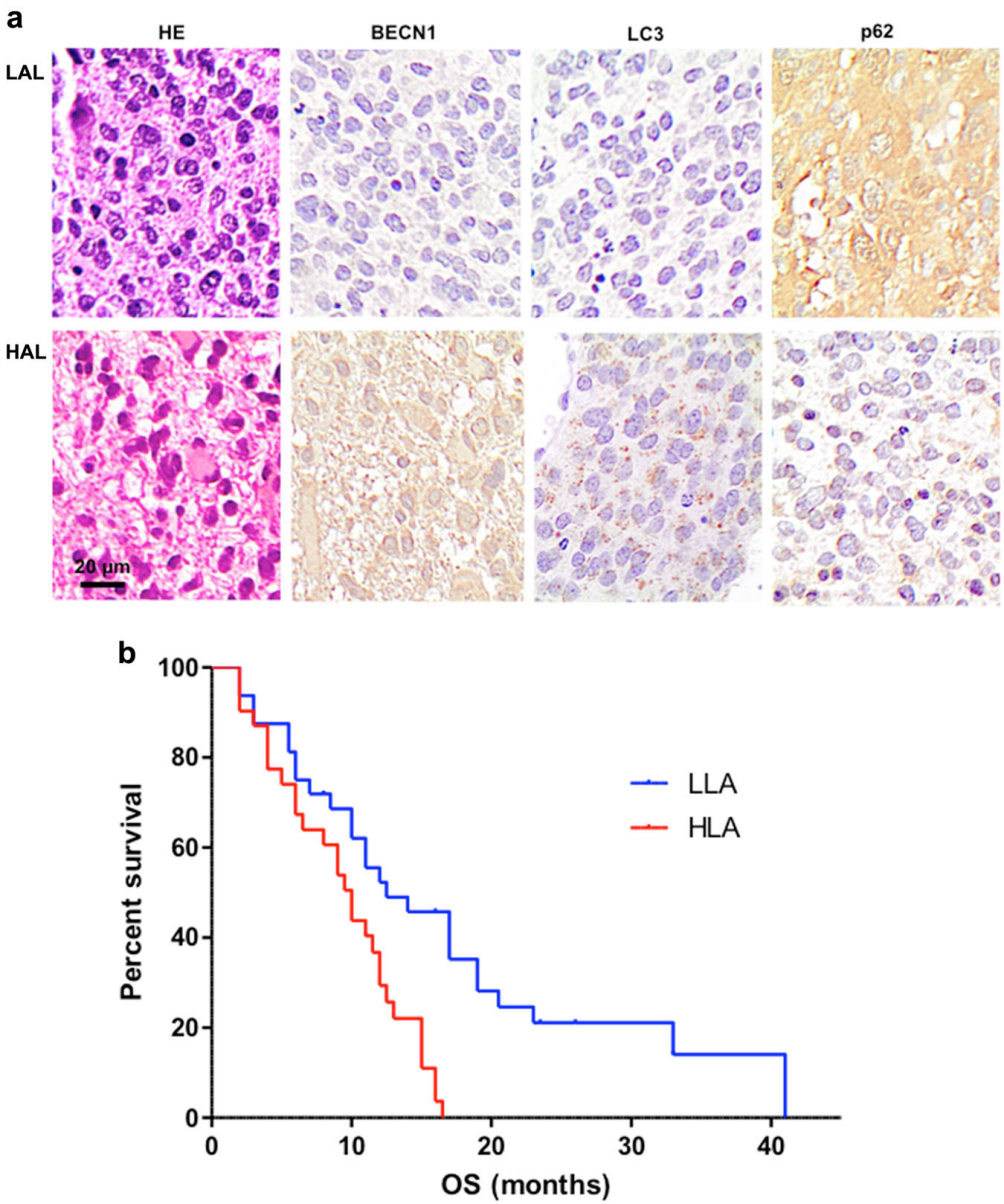
Ex vivo analysis of autophagic markers in GBM samples and patients’ overall survival. **a** Histological sections from GBM patients stained with hematoxylin/eosin or after immunostaining for BECN1, LC3, or SQSTM1 (p62). Avidin-biotin-peroxidase complex method lightly counterstained with hematoxylin. Original magnification 400×. **b** Kaplan-Meier curve generated by GraphPad software. Blu line indicates GBM patients with low levels of autophagy (LAL), while red line indicates GBM patients with high levels of autophagy (HAL). Statistical analysis: *p* = 0.0012; HR 0.3634; 95% CI from 0.1967 to 0.6715

The role of autophagy in cancer onset and progression has been considered as a critical factor^18^. On this basis, three main markers of autophagy were evaluated: Beclin 1 (BECN1), LC3-II, and p62. As stated by literature^19^, BECN1 interacts with either BCL-2 or PI3k class III, playing a critical role in the regulation of autophagy. The microtubule-associated protein 1A/1B-light chain 3 (LC3) is a soluble protein that is distributed ubiquitously in mammalian cells. The increased expression of LC3-II has been associated with increased autophagic process. As concerns the ubiquitin-binding protein p62, it has been suggested it may function as an autophagosome cargo protein. Since p62 accumulates when autophagy is inhibited, p62 may be used, together with LC3-II, as a marker to study autophagic flux. These paradigmatic markers of autophagy were evaluated in slices obtained from 63 GBM specimens by immunohistochemistry. Two different groups were detectable characterized by high levels of LC3 and low levels of p62 (high autophagic levels, HAL, lower panels in Fig. 1a) or, conversely, low levels of LC3 and high levels of p62 (low autophagic levels, LAL, upper panels in Fig. 1a). We found that the OS of patients with low levels of autophagy (32 subjects) was significantly higher (even more than double) than that of those with high levels of autophagy (31 subjects, *p* = 0.0012; HR 0.3634; 95% CI from 0.1967 to 0.6715). These data are reported in Fig. 1b.

### Basal levels of autophagy and in vitro susceptibility of GSCs to TMZ

**Fig. 2:**
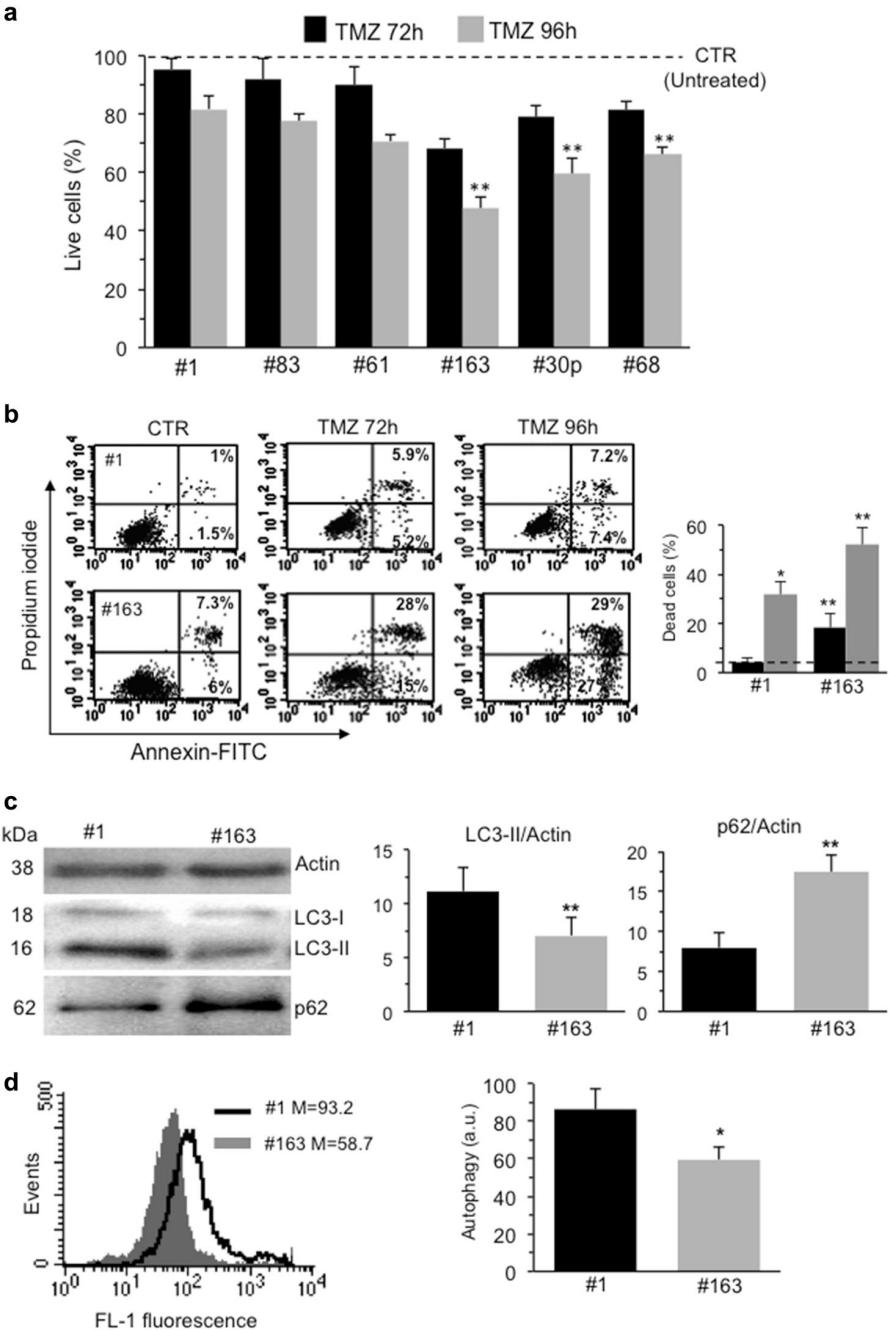
Basal levels of autophagy and in vitro susceptibility of GSCs to TMZ. **a** FACS analysis after staining with calcein-AM (which is retained in the cytoplasm of live cells) of six GSC lines isolated from patients with GBM untreated (dashed line) or treated in vitro with 450 μM TMZ for 72 h and 96 h. Values in ordinate represent the percentage of calcein-positive cells. Results, obtained from four independent experiments, were reported as means ± SD. **b** FACS analysis after double staining with AnnexinV⁄Propidium iodide. Dot plots from a representative FACS experiment are shown. Numbers represent the percentages of annexin V-positive cells (bottom right quadrant) or AnnexinV⁄Propidium iodide double positive cells (upper quadrant). On the right, bar graph showing results obtained from four independent experiments, reported as means ± SD. **c** Western blot analysis using anti-LC3 and anti-SQSTM1 (p62) antibodies. Loading control was evaluated using anti-actin antibody. A representative experiment among three is shown. Bar graphs on the right show densitometric analysis. Results represent the mean ± SD from three independent experiments. **d** Flow cytometry analysis of autophagy in GSC#1 (empty curve) and GSC#163 (full gray curve) performed with a Cyto-ID Autophagy Detection kit. Numbers represent the median fluorescence intensity. A representative experiment among three is shown. On the right, bar graph showing results obtained from three independent experiments, reported as means ± SD. (*) and (**) indicate *p* < 0.05 and *p* < 0.01 *vs*. GSC#1, respectively

Many in vitro and in vivo studies have demonstrated that GSCs are highly resistant to most of the common therapies, including radiation and TMZ^20–22^. We evaluated the sensitivity to TMZ of six out of our collection of GSCs after in vitro treatment with a clinically relevant dose of the drug^23^ by using Calcein-AM to evaluate cell viability. Our data confirmed a general resistance to TMZ of all the tested GSCs after both 72 h and 96 h treatment, although with a different degree (Fig. 2a). Among these six GSC lines analyzed, we selected two representative ones, #1 and #163, that showed a significant difference in the sensitivity to TMZ, and performed Annexin V/Propidium iodide staining after both 72 h and 96 h treatment. Cytofluorimetric analysis revealed morphological signatures of apoptosis, i.e. positivity to Annexin V, also confirming a different sensitivity to TMZ between the two GSC lines (Fig. 2b).

It has been shown that autophagy mostly functions as a survival mechanism^24,25^. As shown in Fig. 2c, autophagy was checked by western blot analysis, using anti-LC3 and anti-p62 antibodies and by flow cytometry, using autophagy detection kit. Western blot analysis revealed a band corresponding to LC3-II more pronounced in #1 than in #163. By contrast, the amount of p62 was significantly more abundant in #163 cells (Fig. 2c, left panels). These results were also confirmed by densitometric analyses (Fig. 2c, bar graphs). Accordingly, we found a significant (*p* < 0.05) increase of green fluorescence after staining with autophagy detection kit in #1 in comparison with #163 (Fig. 2d). Thus, the autophagic levels observed in the two cell lines were inversely proportional to their susceptibility to TMZ.

### Modulation of autophagy in TMZ-induced cell death

**Fig. 3:**
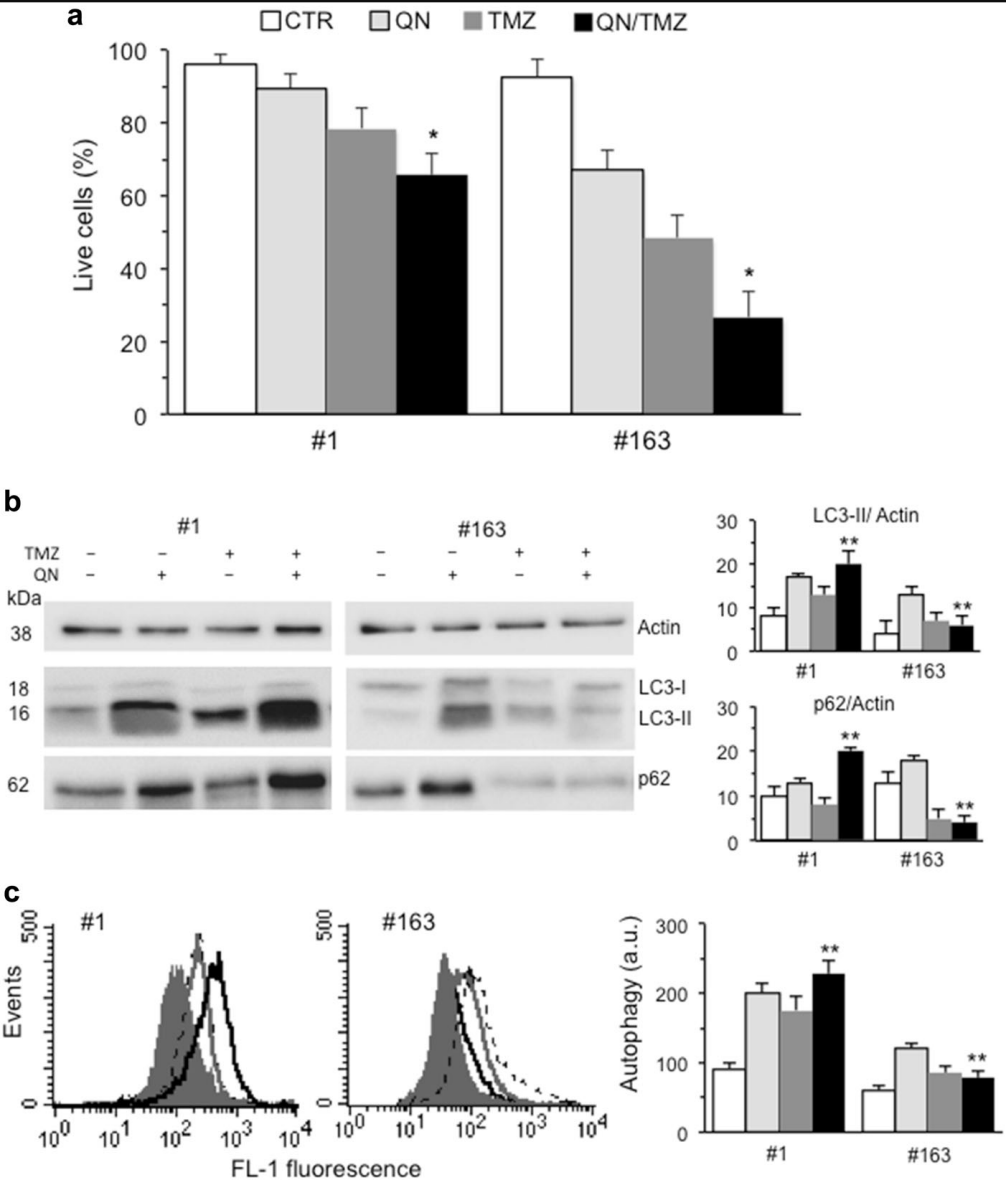
Modulation of autophagy in TMZ-induced cell death: autophagy inhibition by QN. **a** FACS analysis after staining with calcein-AM (which is retained in the cytoplasm of live cells) of GSC#1 and GSC#163 untreated or treated 96 h with 450 μM TMZ, 5 μM QN or their association. Values in ordinate represent the percentage of calcein-positive cells. Results, obtained from four independent experiments performed in duplicate, were reported as means ± SD. **b** Western blot analysis using anti-LC3 and anti-SQSTM1 (p62) antibodies of GSC#1 and GSC#163 untreated (control) or treated with TMZ, QN, or their association. Loading control was evaluated using an anti-actin antibody. A representative experiment among three is shown. Bar graphs on the right show densitometric analysis. Results represent the mean ± SD from three independent experiments. **c** Flow cytometry analysis of autophagy in GSC#1 and GSC#163 performed with a Cyto-ID Autophagy Detection kit. A representative experiment among three is shown. On the right, bar graph showing results obtained from three independent experiments and reported as means ± SD. (*) and (**) indicate *p* < 0.05 and *p* < 0.01, respectively, *vs*. TMZ-treated cells

We modulated autophagic process in both #1 and #163 GSCs by trehalose (TRE), a disaccharide able to induce autophagy *via* an mTOR-independent pathway^26^. We found that co-treatment with TRE and TMZ increased significantly the resistance to this drug in both cell lines, as demonstrated by the increase of cell viability (Supplementary Fig. 1A). In addition, we found that that single treatment with TMZ or TRE was able to induce autophagy in both cell lines, although TRE/TMZ association was significantly more effective (Supplementary Fig. 1B and 1C). Then, we used two drugs, hydroxychloroquine (HCQ) and its derivative quinacrine (QN) able to cross the blood-brain barrier, which are known to inhibit autophagy by raising the lysosomal pH^27^. Differently from HCQ, a moderate cytotoxic effect was observed in QN-treated cell specimens and, according to our hypothesis, we also observed a significant reduction of survival in cells treated with QN/TMZ association (Fig. 3a). The effects induced by QN were well underlined by western blot analysis (Fig. 3b), which revealed an accumulation of LC3-II together with an increase of p62, compatible with a block of autophagic processes in QN-treated GSCs as compared to control cells. Interestingly, in #163 cell line we observed a significant decrease of p62 amount, possibly caused by intracellular degradation events associated with cell death, much higher in this cell line. Flow cytometry evaluation of autophagy substantially confirmed western blot analyses (Fig. 3c). Overlapping results were obtained by using HCQ (Supplementary Figure 2). Data obtained in a further GSC clone (#83) are shown in Supplementary Figure 3.

### In vitro characterization of cell death induced by QN/TMZ association

Then we used inhibitors specific for different types of cell death. GSCs #1 and #163 were therefore treated with: (i) Z-VAD-FMK, an inhibitor of caspase-dependent apoptosis; (ii) Necrostatin 1-s, a specific inhibitor of necroptosis; (iii) CA074-Me or Pepstatin A-Me, cathepsin B and cathepsin D inhibitor, respectively; (iv) calpain inhibitor I, since calpain activity is thought to be essential for the execution of caspase-independent apoptosis in certain experimental models; (v) DFO, an iron chelator able to prevent ferroptosis, and (vi) ferrostatin 1, a lipophilic reducing agent that prevent ferroptosis.

**Fig. 4:**
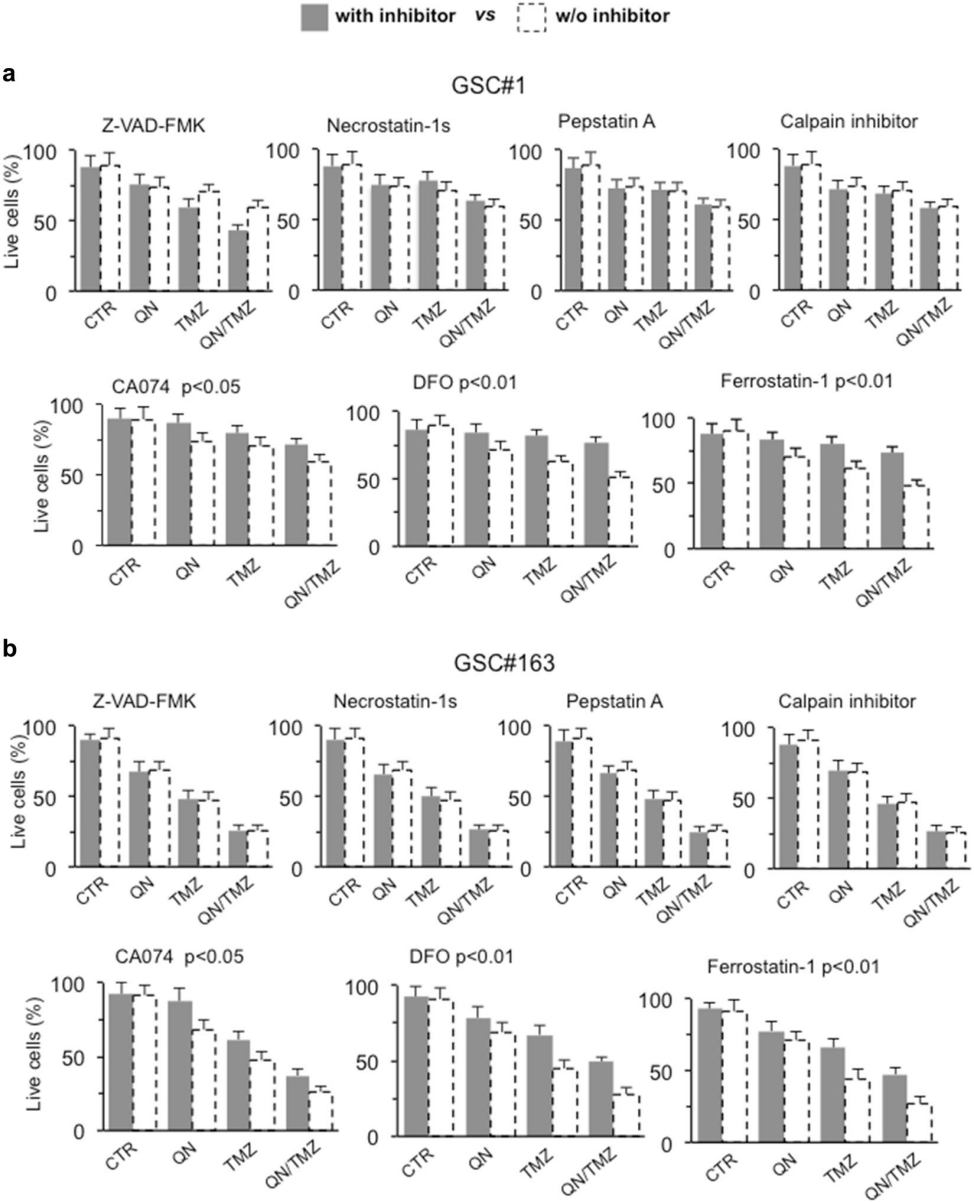
In vitro characterization of cell death induced by QN/TMZ association in GSC#1 and GSC#163. FACS analysis after staining with calcein-AM (which is retained in the cytoplasm of live cells) of **a** GSC#1 and **b** GSC#163 untreated or treated 72 h with 450 μM TMZ, 5 μM QN or their association in the presence or absence of specific inhibitors of different cell death pathways. Values in ordinate represent the percentage of calcein-positive cells. Results are the mean among three independent experiments performed in duplicate and are reported as means ± SD. Where indicated, *p* values are referred to cells treated with TMZ/QN (dashed columns) *vs*. cells underwent the same pharmacological treatments in the presence of the specified death inhibitor

Among the seven inhibitors used, only DFO and ferrostatin 1 were able to significantly prevent cell death, whereas CA074 only partially reduced cell death induced by TMZ, alone or in association with QN, in both cell lines (Fig. 4a, b). These data were also in accordance with our biochemical analyses that did not show any caspase activation (not shown) and seem to suggest that TMZ, alone or in association with QN, induced in GSCs a ferroptotic cell death. In fact, ferroptosis can be suppressed by DFO or ferrostatin 1, but is not affected by apoptosis (e.g., Z-VAD-FMK) or necroptosis (e.g., Necrostatin 1-s) inhibitors^28^.

**Fig. 5:**
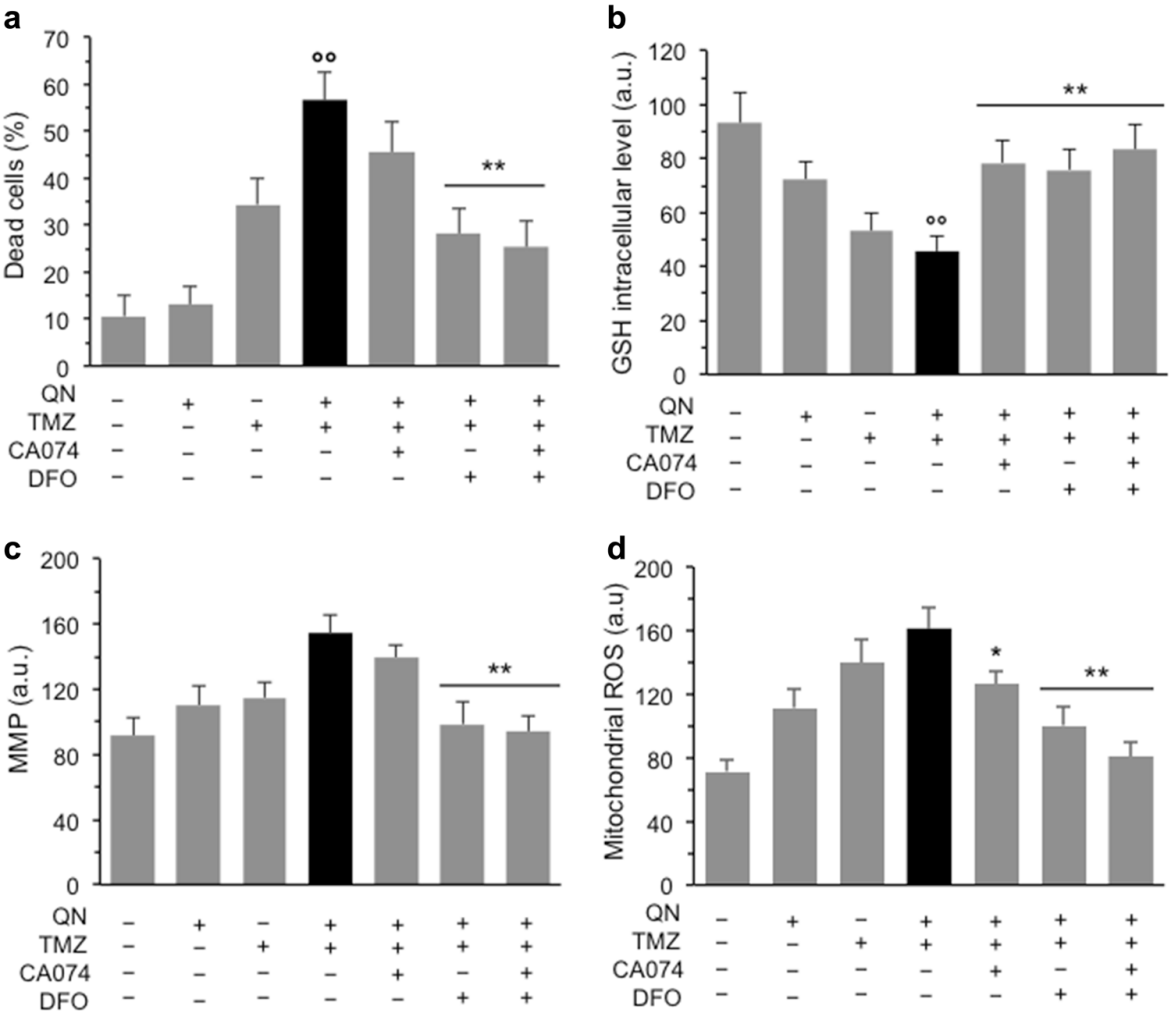
In vitro characterization of cell death induced by QN/TMZ association. Effects of CA074 and ferroptosis inhibitors in GSC#1. **a** FACS analysis after staining with calcein-AM in GSC#1 untreated or treated 72 h with 450 μM TMZ, 5 μM QN or their association in the presence or absence of CA074, DFO, or both. Values in ordinate represent the percentage of dead calcein-negative cells. **b** GSH intracellular content after cell staining with MCB. **c** Mitochondrial membrane potential (MMP) analysis performed by using TMRM. **d** Mitochondrial ROS production by using MitoSOX-red. Bar graphs show results obtained from three independent experiments performed in duplicate and reported as means ± SD. In ordinate, the median fluorescence intensity. (*) and (**) indicate *p* < 0.05 and *p* < 0.01, respectively, *vs*. TMZ/QN-treated cells. (°°) indicate *p* < 0.01 *vs*. untreated samples

When we tested the effect of the combination of the two inhibitors, CA074 and DFO, on cell line #1, the most resistant to TMZ, we found that while CA074 or DFO alone reduced QN/TMZ-induced cell death of about 30%, their combination was able to reduce cell death of about 50% (Fig. 5a), suggesting that different types of death contribute to the cytotoxic effects induced on GSCs by QN, TMZ, and their combination: caspase-independent cathepsin B-dependent apoptosis and ferroptosis. Then, we also evaluated by flow cytometry a series of redox and mitochondrial parameters in the presence or absence of CA074 and/or DFO. We found that: (i) cell production of hydrogen peroxide and superoxide anion were non significantly altered among different experimental samples (data not shown), but, by contrast, (ii) the GSH intracellular levels were reduced by TMZ and more dramatically by QN/TMZ association. This reduction of GSH was considerably prevented both by CA074 and DFO (Fig. 5b). As far as mitochondrial parameters were concerned, we did not observe a drop of membrane potential typical of apoptosis, but rather a state of mitochondrial hyperpolarization in cells treated with TMZ and QN, alone or in association, that was counteracted significantly by DFO, but not by CA074 (Fig. 5c). In addition, we also found a moderate increase of mitochondrial reactive oxygen species (ROS) in cells treated with QN and TMZ alone that became more significant in cells treated with both drugs (Fig. 5d). This drug-induced mitochondrial ROS production was more efficiently prevented by DFO (*p* < 0.01 *vs*. QN/TMZ) than by CA074 (*p* < 0.05 *vs*. QN/TMZ).

### In vitro evaluation of ferroptosis markers

**Fig. 6:**
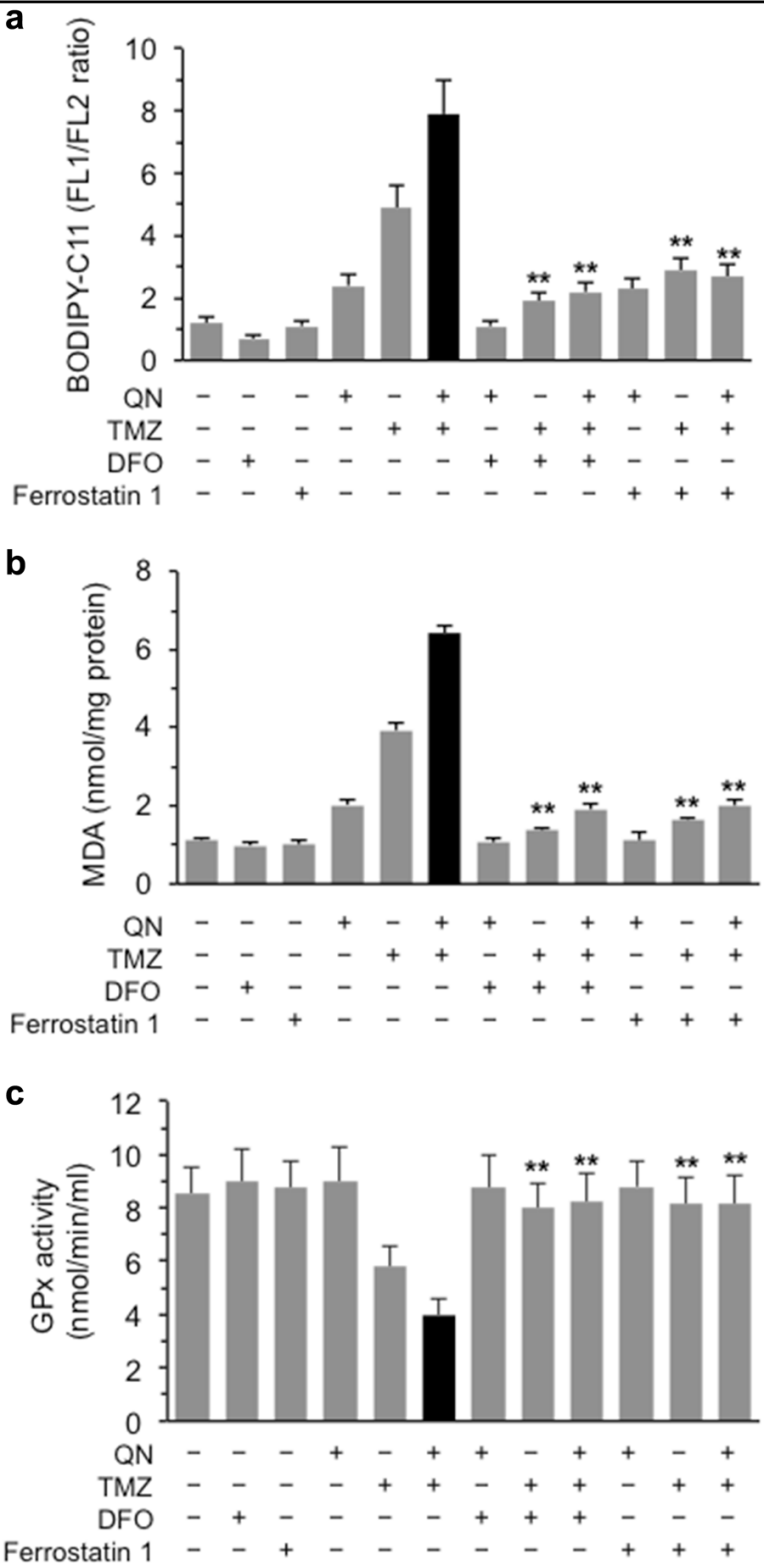
In vitro evaluation of ferroptosis markers. **a** Flow cytometry evaluation of lipid ROS levels by using BODIPY-C11 dye. **b** Colorimetric measurement of MDA production and **c** spectrophotometric evaluation of GPx activity in GSC#1 untreated or treated with TMZ, QN, or their association in the presence or absence of the ferroptosis inhibitors DFO or Ferrostatin 1. Results obtained from two independent experiments performed in triplicate are reported as means ± SD. (**) indicate *p* < 0.01 *vs*. TMZ/QN-treated cells

Ferroptosis involves metabolic dysfunction that results in the production of lipid ROS^29^. Thus, we evaluated either lipid ROS intracellular level by flow cytometry using BODIPY-C11 dye or the most important end-product of lipid peroxidation, malondialdehyde (MDA), believed to be the primary source of damage in cell death due to ferroptosis, which accumulation in cells is considered a good marker of ferroptosis. Cell staining with BODIPY-C11 revealed an accumulation of lipid radicals in cells treated with TMZ alone or in association with QN and, to a lesser extent, also in cells treated with QN alone. This increase in lipid radicals was significantly prevented by both DFO and ferrostatin 1 (Fig. 6a). According with this, in samples treated with the QN/TMZ association we also observed an accumulation of MDA that was more than doubled compared to the single treatment with TMZ (Fig. 6b). Either DFO or ferrostatin 1 were able to significantly prevent lipid peroxidation, as demonstrated by measuring lipid radicals by BODIPY-C11 dye, and the consequent accumulation of MDA (Fig. 6a, b).

Glutathione peroxidase 4 (GPX4) is an essential regulator of ferroptotic cancer cell death by virtue of its ability to scavenge hydrogen and lipid peroxides under oxidative stress^30^. As shown in Fig. 6c, we found that cell treatment with TMZ, alone or in association with QN, reduced the activity of GPx and that either DFO or ferrostatin 1 were able to significantly prevent this drop of GPx activity.

### Cytotoxic activity of TMZ and QN, alone or in association, in neutral and acidic pH

**Fig. 7:**
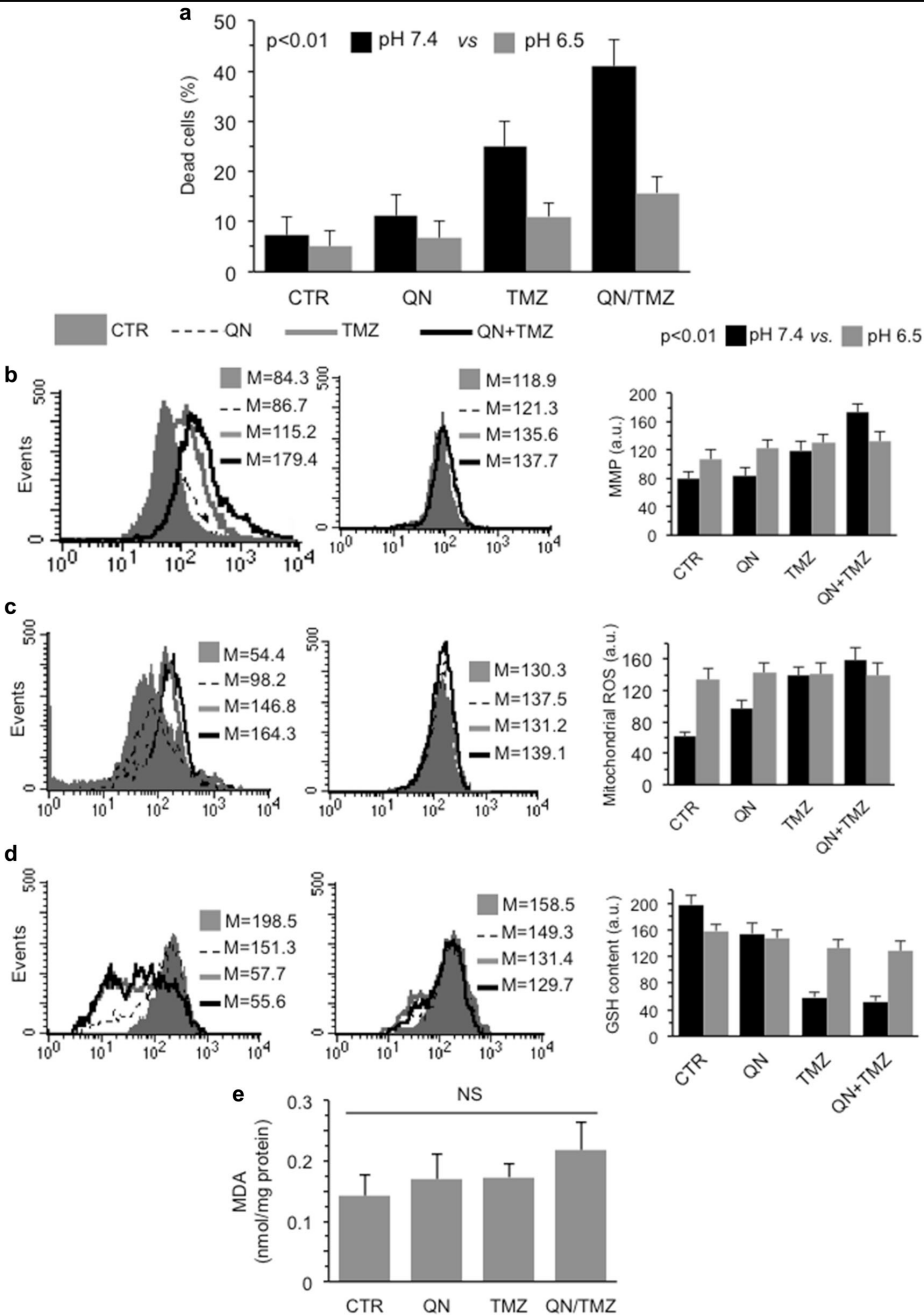
Cytotoxic activity of TMZ and QN, alone or in association, in acidic medium. FACS analysis of different cellular parameters carried out in GSC#1 treated 72 h with 450 μM TMZ, 5 μM QN or their association in neutral (pH 7.4, left panel) or acidic (pH 6.5, right panel) medium. **a** Cell death evaluation after staining with calcein-AM. In ordinate, the percentage of dead cells (negative to calcein-AM). **b** Mitochondrial membrane potential (MMP) analysis performed by using TMRM. **c** Mitochondrial ROS production by using MitoSOX-red. **d** GSH intracellular content after cell staining with MCB. In ordinate, the median fluorescence intensity. *p* < 0.01 TMZ/QN at pH 7.4 *vs*. QN/TMZ at pH 6.5. **e** Colorimetric measurement of MDA in GSC#1 cells untreated or treated 72 h with 450 μM TMZ, 5 μM QN or their association. Results obtained from three independent experiments performed in duplicate were reported as means ± SD

Since it has been reported that CQ was unable to block the autophagic flux at acidic pH^31^, and considering that acidic pH is an important feature of tumor microenvironment and a major determinant of tumor progression, we evaluated the cytotoxic effects of QN, TMZ, and their association in GSC#1 cultured at pH 6.5. We found that acidic pH inhibited significantly cytotoxic effect induced both by TMZ and QN, alone or in combination (Fig. 7a). In addition, we also found that the increase of mitochondrial membrane potential (Fig. 7b) and of mitochondrial ROS production (Fig. 7c) induced by TMZ, alone or in combination with QN, which were associated with the reduction of intracellular GSH (Fig. 7d), were significantly prevented at pH 6.5. According with this, we did not observe any accumulation of MDA neither in cells treated with TMZ or with QN, alone or in combination (Fig. 7e).

### In vivo effect of TMZ and QN, alone or in association, on the growth of GSC#1 brain xenografts

**Fig. 8:**
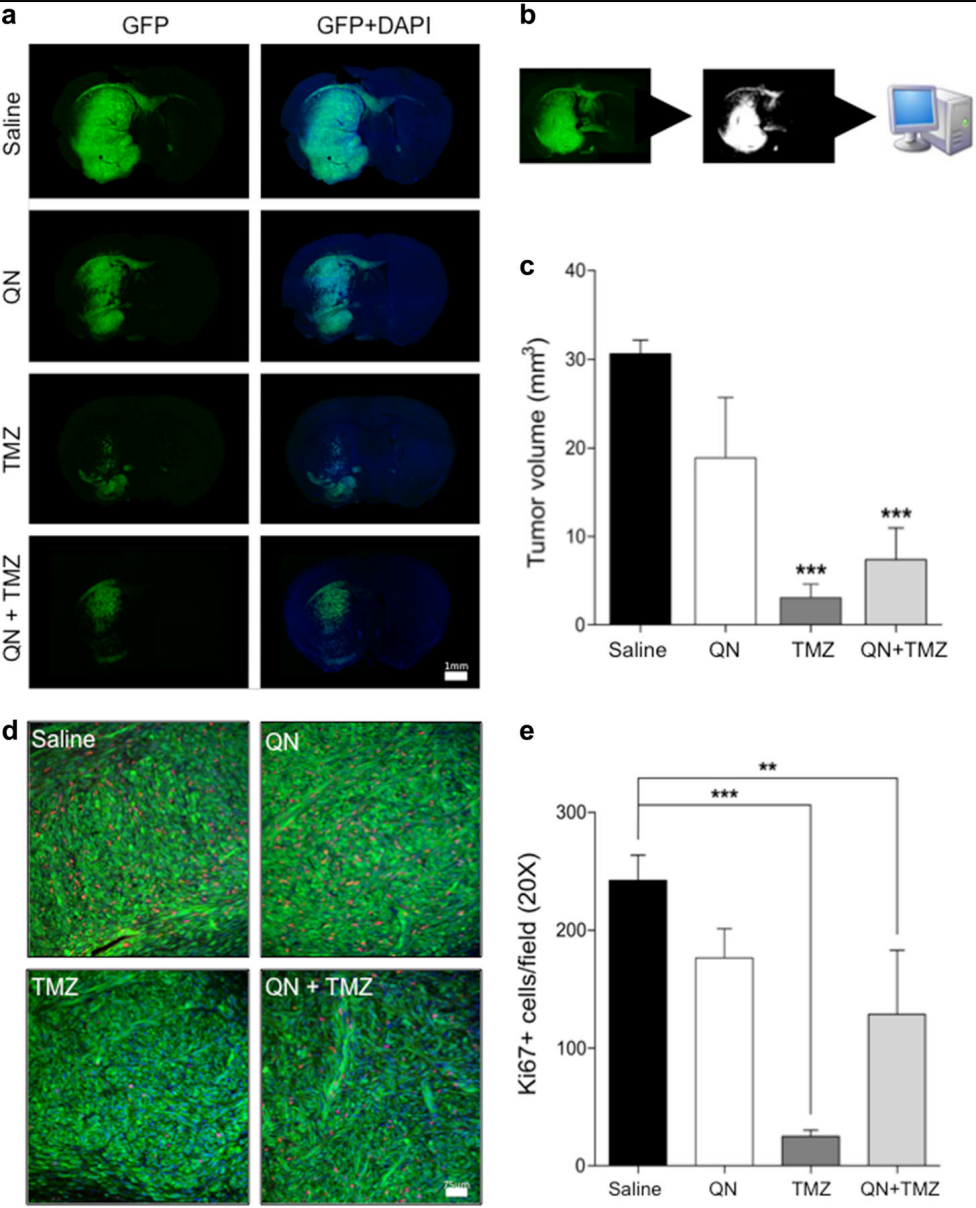
In vivo effect of TMZ and QN, alone or in association, on the growth of GSC#1 brain xenografts. Effects of QN, TMZ, and combined TMZ *plus* QN on the growth of intracerebral GFP-expressing GSC#1 xenografts. **a** Photomontages of representative coronal sections of the mouse brain illustrating the site of cell injection and brain invasion by 16 weeks after grafting. Saline-treated control mice developed large infiltrating tumors in the brain hemisphere homolateral to the grafting side. Tumor cells spread contralaterally along the corpus callosum and anterior commissure. Treatment with QN did not change the growth pattern of brain tumors. Conversely, both TMZ-treated mice and mice treated with combined TMZ *plus* QN harbored smaller tumors with remarkable reduction of brain infiltration in the grafted hemisphere and inter-hemispheric pathways as well. Scale bar, 1 mm. **b** Schematic drawings showing the method used for automated demarcation of brain infiltration and for calculating the volume of the brain region invaded by the GFP-expressing GSC#1. **c** Diagrams showing the volume of the brain region invaded by the GFP-expressing GSC#1 (***p < 0.0001). **d** Representative immunostaining with Ki67 for assessment of cell proliferation. Scale bar, 75 μm. **e** Proliferation of tumor cells was significantly reduced both in TMZ-treated mice and mice treated with combined TMZ *plus* QN (***p < 0.0001; *p < 0.05)

Intracerebral injection of GSC#1 in immunodeficient mice generates highly infiltrative tumor xenografts that closely mimic the behavior of malignant gliomas^32–34^. Stable GFP-expressing GSC#1 was grafted into the striatum of NOD-SCID mice. Twelve weeks after grafting, the mice were treated for 3 weeks either with saline, or with QN alone, or with TMZ alone, or with TMZ *plus* QN. Analysis of tumor volumes on fluorescent microscopy of seriated brain sections showed that both mice treated with TMZ and those treated with combined TMZ and QN harbored significantly smaller tumor than saline-treated mice (Fig. 8a–c). There were not significant differences in tumor volume between mice treated with QN alone and saline-treated controls, or between mice treated with TMZ alone and those treated with TMZ *plus* QN, suggesting that QN did not increase the anti-tumor effect of TMZ.

Analysis of tumor cell proliferation, as assessed by Ki67 labeling index, showed that as compared with saline-treated tumors, those treated with TMZ alone showed a highly significant decrease of cell proliferation, whereas proliferation was inhibited to a less extent in tumors treated with TMZ *plus* QN (Fig. 8d, e). QN alone did not decrease proliferation significantly.

**Fig. 9:**
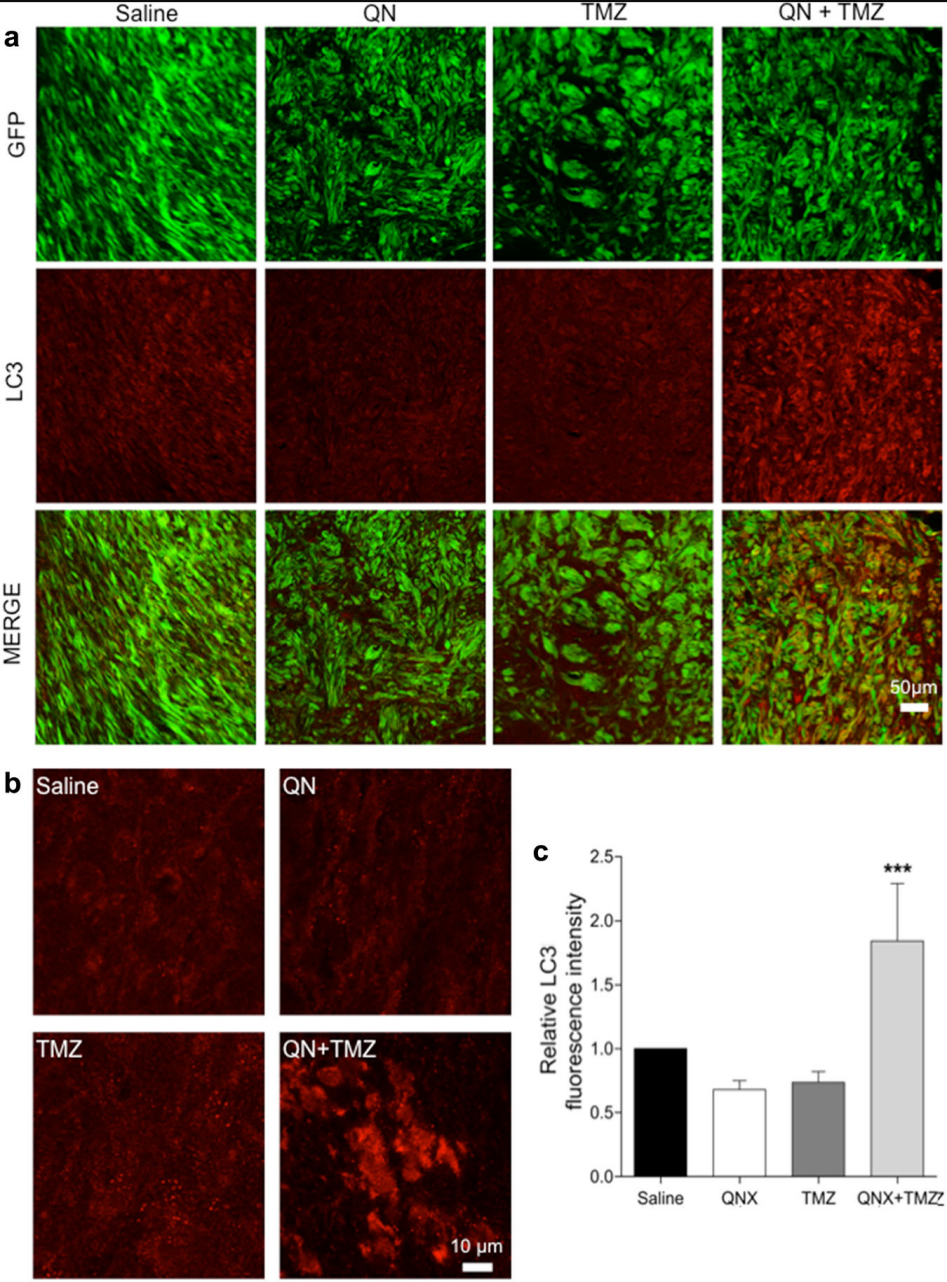
Anti-LC3 immunostaining for assessment of autophagy in GSC#1 brain xenografts treated with QN, TMZ, and combined TMZ *plus* QN. **a** Representative images of brain sections through the tumor xenografts. Scale bar, 50 μm. **b** Examples of images used for automated assessment of fluorescence intensity acquired with fixed filter settings. Scale bar, 10 μm. **c** Diagrams comparing LC3 fluorescence intensity of brain xenografts. Tumors treated with TMZ *plus* QN showed a significant increase of LC3 expression (***p < 0.0001)

To test our working hypothesis, i.e., that inhibition of autophagy by QN might sensitize GSC#1 to TMZ, we assessed LC3 expression in the tumor xenografts. Computerized analysis of immunofluorescence intensity showed that the expression of LC3 was significantly increased only in tumors treated with the combination of TMZ and QN (Fig. 9). There were no significant changes of LC3 expression and distribution in GSC#1 xenografts treated with TMZ or QN alone as compared with saline-treated controls. Taken together, our in vivo results show that adjunctive treatment with QN does not enhances the antitumor effect of TMZ, as assessed by tumor volume and cell proliferation analyses.

Histological analysis of visceral organs revealed that treatment with TMZ and with TMZ *plus* QN did not determine kidney modifications (data not shown), which resulted in thickening of the alveolar wall of lungs (Supplementary Fig. 4). The liver of mice treated with TMZ or with combined TMZ and QN showed an activation of Kupfer’s cells associated with depletion of cellular and nuclear glycogen (Supplementary Fig. 5). Finally, the spleen of mice treated with QN or with combined QN and TMZ showed an increase of fibrotic tissue associated with activated macrophages and prevalent erythropoiesis (Supplementary Fig. 6).

## Discussion

The aggressiveness of GBM partly relies on a subpopulation of tumor cells, termed as GSCs, which show functional properties, like multipotency and self-renewal, similar to those of neural stem cells^35^. GSCs reside in vascular niches in close contact with brain endothelial cells, which may regulate GSC self-renewal, determine cell fate, and protect these cells from chemo- and radiation therapies^36^.

Autophagy has been found enhanced in human gliomas as compared to normal brain. In particular, some lines of evidence suggest that glioma cells are rather prone to autophagy induction, whereas they appear as resistant to apoptosis triggering^37^. However, there are only few data concerning the diagnostic and clinical relevance of the autophago-lysosomal network in human glioma tissue. Our ex vivo analyses add some insight on this matter since human GBM tissue specimens revealed that survival was significantly higher in patients with low levels of autophagy than in those with high levels of autophagy. This seems to confirm that the hypothesized role of the cytoprotection mechanism exerted by the autophagic flux could represent an important survival strategy of GBM cancer cells^38^.

Chemotherapeutic drugs, including TMZ, can exert their therapeutic effects by triggering autophagy^39^. Accordingly, autophagy-mediated apoptosis stimulating agents, such as Δ9-tetrahydrocannabinol^40^ and oncolytic adenovirus CRAd-Surivin-pk7^41^, combined with TMZ, were reported to strongly reduce the growth of glioma xenografts, suggesting that the combined administration of TMZ and autophagy inhibitors could be therapeutically exploited for the management of GBM. Furthermore, it has been shown that inhibition of autophagy at early steps of cancer growth can reduce the effects of chemotherapeutic treatment with TMZ, whereas inhibition of autophagy at later stages sensitized glioma cells to TMZ^9^. Results obtained in our in vitro experiments on GSCs with different degrees of resistance to TMZ confirm that pharmacological inhibition of autophagy results in accumulation of autophagic vacuoles, favoring the cytotoxicity of TMZ. Indeed, we observed a significant reduction of survival in cells treated with QN/TMZ association. This synergistic activity was more pronounced in GSCs with a higher susceptibility to TMZ.

Unfortunately, the association of QN/TMZ did not produce a synergistic effect on our mouse model of brain xenografts. Accordingly, QN alone was not able to inhibit autophagy in vivo, as demonstrated by LC3 amount in GSC#1 xenografts treated with QN that did not differ from saline-treated controls. The level of LC3 was significantly increased in tumors treated with the combination of QN and TMZ only.

Several mechanisms could lead to the disparity of the in vitro and in vivo results obtained in our study with GSCs. One of these could be referred as to acidosis. The in vitro data seem to confirm this hypothesis, since we found that acidic pH of the cell medium (pH 6.5) significantly decreased the cytotoxic effect of the association QN/TMZ. Although the inhibition of autophagy is currently being evaluated as a novel tool in anticancer therapies, our observations seem to suggest that drugs that target autophagy could only partially modify the tumor microenvironment in the acidic regions, predicting possible limitations in efficacy of these drugs in antitumor therapy. In fact, strategies aimed at buffering microenvironment pH before administration of standard chemotherapy have been proposed by both preclinical^42^ and clinical studies [Clinical trials NCT01198821]^43^. Therefore, the development of drugs capable of modulating autophagy in acidic microenvironment, which often characterizes tumors, would be desirable.

Treatment of GSCs with QN and TMZ induced a cell death pathway characterized by phosphatidylserine flipping at the cell surface, typical of apoptosis. However, at variance, this cell death process appeared as independent from caspase activity (as also confirmed by the inefficiency of z-VAD in preventing QN/TMZ induced cell death). Further forms of cell death, other than apoptosis, have been discovered in the last years. One of these is called necroptosis that represents a programmed form of necrosis^28,44^. However, although the induction of necroptosis was found to be an effective strategy to kill the glioma cells resistant to apoptosis^45,46^, in our experimental model we did not observe any modulation of RIP1 and RIP3, two molecules that auto- and trans-phosphorylate each other leading to the formation of the necrosome. Accordingly, necrostatin, a necroptosis inhibitor, was unable to inhibit cell death induced by QN/TMZ. Conversely, GSC death was significantly prevented by using CA074, an inhibitor of cathepsin cystein protease, particularly cathepsin B, able to hinder cathepsin-mediated cell death, and DFO, an iron chelator able to prevent ferroptosis, a programmed cell death dependent on iron and characterized by the accumulation of lipid peroxides. As concern the first, the cathepsin-mediated cell death, our data are in line with those provided by other authors, demonstrating that inhibitors of autophagy, i.e., CQ and the phytochemical thymoquinone, could mediate lysosome permeability, resulting in leakage of cathepsin B into the cytosol where it can act as mediator of cell death^47^. As far as ferroptosis is concerned, it represents an emerging and recently discovered type of regulated cell death occurring through Fe(II)-dependent lipid peroxidation^48–51^. A very recent review on different types of cell death proposes to define ferroptosis as a form of regulated cell death initiated by oxidative perturbations of the intracellular microenvironment inhibited by iron chelators and lipophilic antioxidants^28^, but is not affected by apoptosis or necroptosis inhibitors^51^. Monitoring oxidative stress in ferroptotic cells showed an increase in lipid peroxidation without any increase of ROS such as superoxide^52^. Accordingly, in GSCs treated with QN/TMZ we did not observe any increase of ROS production, i.e., superoxide anion or hydrogen peroxide. At variance, a significant increase of mitochondrial ROS generation, paralleled by an increase of mitochondrial membrane potential, i.e., a mitochondrial membrane hyperpolarization, was observed. It has recently been reported that mitochondria may have a central role in ferroptosis^53^, as also demonstrated by the fact that cell treatment with mitochondria-targeted antioxidants, capable of preventing the oxidation of cardiolipin, strongly prevented ferroptotic cell death^53^. In light of this, the hyperpolarization of mitochondrial membrane and the increase of mitochondrial ROS found in GCSs treated with TMZ, alone or together with QN, could be explained as a consequence of a mitochondrial damage due to peroxidation of membrane lipids, a hallmark of ferroptosis. These data are also in accord with a number of recent works identifying lipid peroxidation as primary determinant of ferroptosis^48,49,54^.

Several recent studies have confirmed the pivotal role of ferroptosis in killing tumor cells and suppressing tumor growth. In fact, ferroptosis induced by erastin improved the efficacy of chemotherapy when administered together with chemotherapeutic drugs, including TMZ^55^. In particular, in this study^55^, GSH levels were found to be strictly associated with the sensitivity of GBM cells to TMZ, whereas drug-inducible ROS level was decreased in GBM cells resistant to TMZ^55^. These observations are in line with the results reported in our study on GSCs. Erastin and other inducers of ferroptosis could be specifically useful as agents able to target certain cancer stem cell populations that have shown an intrinsic resistance to radiotherapy-induced, ROS-mediated cell death owing a high expression of enzymes of the glutathione synthesis pathway^55^. Accordingly, in a very recent paper^56^, it has been reported a pronounced effect of ironomycin, an inducer of ferroptosis, against CSCs derived from breast human mammary epithelial cells. These findings could directly implicate that iron homeostasis could represent a key regulator of the survival or death of CSCs^57^. Accordingly, in GSCs we detected an activation of cathepsin B that may be due to lysosome membrane permeabilization, lipid peroxidation, and decreased endogenous levels of the reduced form of glutathione, which is consistent with the induction of ferroptotic cell death.

Autophagy can contribute to ferroptosis by degradation of ferritin in cancer cells, but more recent studies demonstrated that activation of autophagy was required for the induction of ferroptosis also in normal cells^58^. Although the relationship between autophagy and ferroptosis at the genetic level remains unclear, different groups recently indicated ferroptosis as an autophagic cell death process^59,60^. According with our data, it has also been observed that treatment with lysosome inhibitors decreased ferroptotic cell-death-associated ROS burst. These lysosome inhibitors could partially prevent intracellular iron delivery by attenuating intracellular transport of transferrin or autophagic degradation of ferritin^58^.

Altogether, these findings underscore the relevance of ferroptotic cell death for cancer stem cells and pave the way towards an improvement of the research on the mechanisms of ferroptosis. Its induction in cancer stem cells could in fact represent an innovative therapeutic strategy aimed at eradicating GBM malignancy.

## Materials and methods

### Immunohistochemistry of GBM tumor specimens

Immunohistochemistry was performed on deparaffinized sections using the avidin-biotin-peroxidase complex methods (ABC-Elite kit, Vector, Burlingame, CA) with freshly made diaminobenzidine as a chromogen as elsewhere described^61^. The expression of LC3 was assessed with mouse monoclonal anti-LC3 antibody (MBL Int. Corporation, Woburn, MA); the expression of p62 was detected with anti-p62/SQSTM1 antibody produced in rabbit (Sigma-Aldrich Inc., Saint Louis, MO), and the expression of BECN1 was detected with an antibody produced in rabbit (Thermo Fisher, Eugene, OR). Endogenous biotin was saturated by biotin blocking kit (Vector). For antigen retrieval, paraffin sections were microwave-treated in 0.01 M citric acid buffer at pH 6.0 for 10 min. GBMs were classified as high autophagic when immunostaining for LC3 labeled more than 50 percent of cells, and p62 was reduced as compared to endothelial cells. Conversely, negative immunostaining for LC3 and strong expression of p62 identified low autophagic GBM. The immunohistochemical results were evaluated independently by two pathologists (LML and MM), who were unaware of the clinical data.

### GSC cultures

GSCs were isolated through mechanical dissociation of the tumor tissue collected from adult patients with GBM tumors (WHO grade IV) undergoing complete or partial surgical resection at the Institute of Neurosurgery, Catholic University School of Medicine in Rome. Informed consent was obtained from the patients before surgery. Single cell suspension was cultured in a serum-free medium supplemented with epidermal growth factor and basic fibroblast growth factor as previously described^32–34^. The in vivo tumorigenic potential of GSCs was assayed by intracranial cell injection in immunocompromised mice where GSCs were able to recapitulate the human tumor in antigen expression and histological tissue organization.

### In vitro cell treatments

GSCs were mechanically dissociated and plated at a density of 10 × 10^4^ six-well microtiter plates. After 16 h, GSCs were treated for 72 h and 96 h.

The following chemicals and drugs were used: 450 μM Temozolomide (TMZ, Cayman Chemical Inc., Ann Arbor, MI), 10 μM z-VAD-FMK (Enzo Life Sciences, Rome, Italy), 5 μM CA074 (Chemicon International, Inc), 100 mM Trehalose (TRE, Sigma-Aldrich), 5 μM Quinacrine (QN, Sigma-Aldrich), 30 μM hydroxychloroquine (HCQ, Sigma-Aldrich), 10 μM calpain inhibitor I, 100 μM Necrostatin-1 (Enzo Life Sciences) 20 μM pepstatin A (Sigma-Aldrich), 100 μM deferoxamine (DFO, Sigma-Aldrich), and 20 μM ferrostatin 1 (Sigma-Aldrich). Compounds were dissolved in DMSO or in serum-free medium. Samples treated with vehicle alone were considered as control.

### Cell death and viability

ATP levels were measured as a surrogate of cell viability using the CellTiter-Glo™ (Promega Inc., Madison, WI) following the manufacturer’s instructions. The mean of the raw luminescence values from triplicate wells treated with vehicle alone (m*L*_*C*_), was used as reference to interpolate percent viability from wells treated with drugs (*V*_*D*_), using the following formula: *V*_*D*_ = (*L*_*D*_/m*L*_*C*_)*100 as previously described^62^. Quantitative evaluation of apoptosis was performed by flow cytometry after double staining using fluorescein FITC-conjugated Annexin V and 5 μg/ml Propidium iodide 10 min at room temperature, and analyzed by fluorescence-activated cell sorting (FACS) in the FL1 and FL2 channels to determine the percentage of dead cells. Cell viability was evaluated by staining control and treated GSCs with 5 μm calcein-AM (Thermo Fisher) at 37 °C for 30 min.

### Western blot analysis

Control and treated GSCs were suspended in 1 ml of lysis buffer, containing 1% Triton X-100, 10 mM Tris-HCl (pH 7.5), 150 mM NaCl, 5 mM EDTA, 1 mM NaVO4 and 75 U of aprotinin and allowed to stand for 20 min. The lysate was centrifuged for 5 min at 1300 × g to remove nuclei and large cellular debris. Lysate obtained as above, were subjected to sodium-dodecyl sulphatepolyacrilamide gel electrophoresis (SDS-PAGE). The proteins were electrophoretically transferred onto polyvinilidenedifluoride (PVDF) membranes (Bio-Rad, Hercules, California, USA) and probed with: anti-LC3 antibody (MBL), anti-P62 antibody (Sigma-Aldrich) or rabbit monoclonal anti-caspase 3 antibody (Invitrogen, Hillsboro, Oregon). Bound antibodies were visualized with horseradish peroxidase (HRP)-conjugated anti-rabbit IgG or anti-mouse IgG (Jackson ImmunoResearch Laboratories, Baltimore Pike West Grove, PA, USA) and immunoreactivity assessed by chemiluminescence reaction, using the ECL Western detection system (Millipore, Darmstadt, Germania). Densitometric scanning analysis was performed by Chemidoc (Bio-Rad).

### Redox parameters

*Intracellular ROS*. After treatments, GSCs were mechanically dissociated and plated at a density of 2 × 10^4^ cells/ml. Then were incubated in Hank’s balanced salt solution, pH 7.4, with dihydrorhodamine 123 (DHR123, Thermo Fisher) in polypropylene test tubes for 15 min at 37 °C (final concentration 10 μM). DHR123 dye freely diffuses into cells and is primarily oxidized by H_2_O_2_ producing green fluorescence. As this oxidation does not occur in dead cells (no green fluorescence), this staining was also used to evaluate cell loss.

*GSH*. GSH intracellular content was assessed by using monochlorobimane (MBC, Molecular Probes). Samples were washed twice in ice-cold PBS and immediately acquired by an LRS II cytometer (Becton and Dickinson, San Jose, CA, USA) equipped with a UVB laser.

*Mitochondrial ROS*. Cells (5 × 10^4^) were incubated with 5 μM MitoSOX (Red Mitochondrial Superoxide Indicator, Thermo Fisher) in complete medium, for 30 min at 37 °C. Cells were then washed in PBS and immediately analyzed on a cytometer.

*Mitochondrial membrane potential*. The mitochondrial membrane potential (MMP) of controls and treated GSCs were studied by using 5–5’,6–6'-tetrachloro-1,1',3,3'-tetraethylbenzimidazol-carbocyanine iodide probe (JC-1, Molecular Probes, Eugene, Oregon, USA). In line with this method, living cells were stained with 10 μM of JC-1. Tetramethylrhodamine ester 1 μM (TMRM, Molecular Probes) was also used to confirm data obtained by JC-1 (not shown).

### Activity of glutathione peroxidase (GPx)

For measuring GPx activity, we used the conventional spectrophotometry method (Cayman Chemical). The basis of the method is oxidation of GSH and the reduction of organic peroxided by GPx, paired with oxidized glutathione (GSSG) recovery by consumption of NADPH and glutathione reductase activity. The oxidation of NADPH to NADP + is accompanied by a decrease in absorbance at 340 nm. The rate of decrease in the absorbance at 340 is therefore directly proportional to the GPx activity in the sample.

### Lipid peroxidation

Analysis of lipid peroxidation was performed by quantification of malondialdehyde (MDA) by using a specific colorimetric kit (BioVision, Milpitas, CA) following manufacturer’s instructions. MDA reacts with thiobarbituric acid (TBA) to generate the MDA-TBA adduct which can be quantified colorimetrically by a spectrophotometer (OD 532 nm). In addition, lipid ROS level was determined also using 5 µM of BODIPY-C11 (Thermo Fisher), a dye with good spectral separation of the non-oxidized (FL2: 595 nm) and oxidized (FL1: 520 nm) forms. Briefly, cells were incubated for 20 min at 37 °C, washed twice with PBS followed by re-suspending in 300 µl of PBS and immediately analyzed on a cytometer. Results were reported as FL1/FL2 ratio.

### Activation of caspases

The activation state of caspases 3/7, 8, and 9 was evaluated using a CaspGLOW fluorescein active caspase staining kit (MBL, Woburn, MA, USA).

### Intracranial implantation of GSCs in immunocompromised mice

Animal experiments were performed in accordance to relevant institutional and national regulations and were approved by the Ethical Committee of the Università Cattolica del Sacro Cuore, Rome. NOD-SCID mice (male; 4–6 week old; Charles River, Italy) were implanted intracranially with 2 × 10^5^ GFP-expressing GSC#1 resuspended in 5 μl of serum-free DMEM [57]. For brain grafting, the mice were anesthetized with intraperitoneal injection of diazepam (2 mg/100 g) followed by intramuscular injection of ketamine (4 mg/100 g). Animal skulls were immobilized in a stereotactic head frame and a burr hole was made 2 mm right of the midline and 1 mm anterior to the coronal suture, and cells were slowly injected using the tip of a 10-μl Hamilton microsyringe placed at a depth of 3 mm from the dura. Twelve weeks after grafting, the mice were randomly assigned to four groups and treated intraperitoneally according to the following protocol.

**Table:** 

Group	*n*	Treatment	Dose	Schedule
I	6	Saline	2 ml	Three times/week x 3 weeks
II	6	Quinacrine (QN)	10 mg/kg^27,63,64^	Five times/week x 3 weeks
III	6	Temozolomide (TMZ)	50 mg/kg	Three times/week x 3 weeks
IV	6	Temozolomide (TMZ) Quinacrine (QN)	50 mg/kg 10 mg/kg	Three times/week x 3 weeks Five times/week x 3 weeks

During treatment, the body weight and neurological status were monitored daily. One week after the end of treatment, the mice were deeply anesthetized and transcardially perfused with 0.1 M PBS (pH 7.4) followed by 4% paraformaldehyde in 0.1 M PBS. The brain was removed, stored in 30% sucrose buffer overnight at 4 °C, and serially cryotomed at 40 μm on the coronal plane. Sections were collected in distilled water, mounted on slides, and cover-slipped with Eukitt. Images were obtained with a Laser Scanning Confocal Microscope (IX81, Olympus Inc., Melville, NY, USA). The cranio-caudal extension of the brain area invaded by GSCs was assessed on serial coronal sections. Then, histological sections 320 μm apart were digitized; on each image, the brain region containing GSCs was demarcated with the cursor and its area calculated by using a commercially available software. To assess the tumor volume, each area of the infiltrated brain was multiplied for the distance to the consecutive digitized section, starting from the tumor epicentre to the cranial and caudal poles of the tumor, and partial volume values were added^65^. Proliferation and autophagy were evaluated by immunostaining with Ki67 (Merck Millipore, Darmstadt, Germany) and LC3B (anti-rabbit; Cell Signaling Tech), respectively. Images were obtained with a Laser Scanning Confocal Microscope (Flouview FV1000, Olympus Inc.). The liver, lung, kidney, and spleen of mice were also assessed by conventional histology.

### Data and statistical analysis

For flow cytometry studies, samples were analyzed with a FACS calibur cytometer (BD Biosciences) equipped with a 488 argon laser and with a 635 red diode laser. At least 20,000 events were acquired. Data were recorded and statistically analyzed by a Macintosh computer using CellQuest software (BD Biosciences). Collected data from in vitro analyses were carried out by ANOVA 2-way testing for repeated samples, using Graph Pad software (Graph Pad, San Diego, CA, USA). All data were verified in at least 3 independent experiments and are reported as means ± standard deviation (SD). For in vivo studies, statistical analysis was performed using Graph Pad-Prism 5 software (Graph Pad Software, San Diego, CA) and MedCalc version 10.2.0.0 (MedCalc Software, Mariakerke, Belgium). Kaplan-Meier survival curves were plotted and differences in survival between groups of patients were compared using the log-rank test (Graph Pad-Prism 5 software, Graph Pad Software). Only values of *p* < 0.05 were considered as significant.

## Electronic supplementary material

Supplementary Figure 1

Supplementary Figure 2

Supplementary Figure 3

Supplementary Figure 4

Supplementary Figure 5

Supplementary Figure 6
