# Supplementary figures and images for: Inhibition of autophagy increases susceptibility of glioblastoma stem cells to temozolomide by igniting ferroptosis
Source: Cell Death Dis. 2018 Aug 6;9(8):841. doi: 10.1038/s41419-018-0864-7 (PMC6079099; doi:10.1038/s41419-018-0864-7)

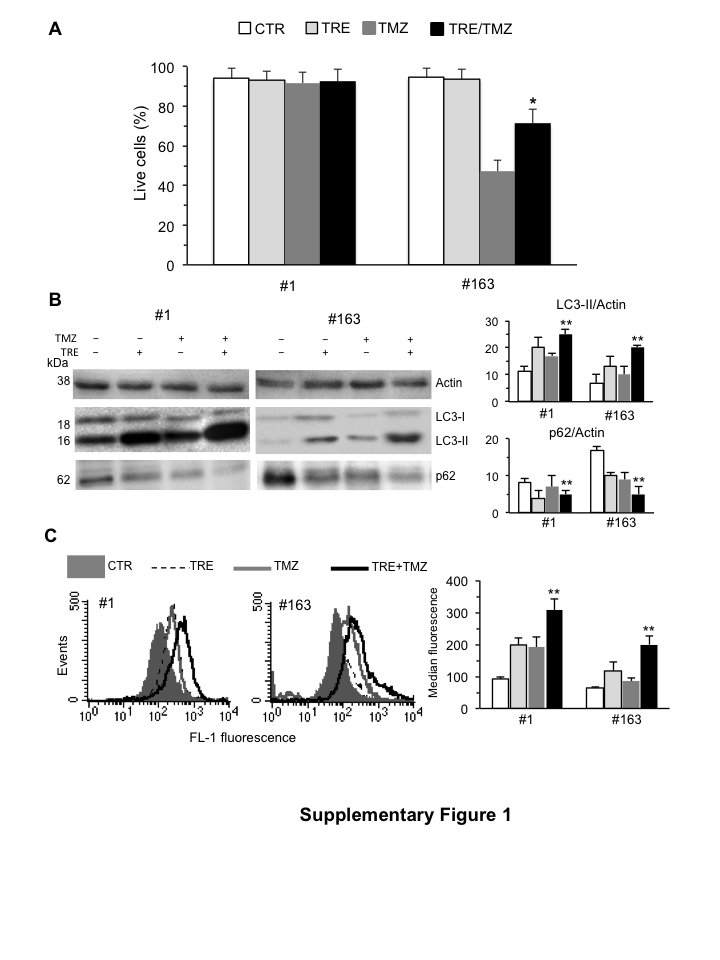

Supplement: Supplementary file 1 — Supplementary Figure 1 [file 41419_2018_864_MOESM1_ESM.tif]

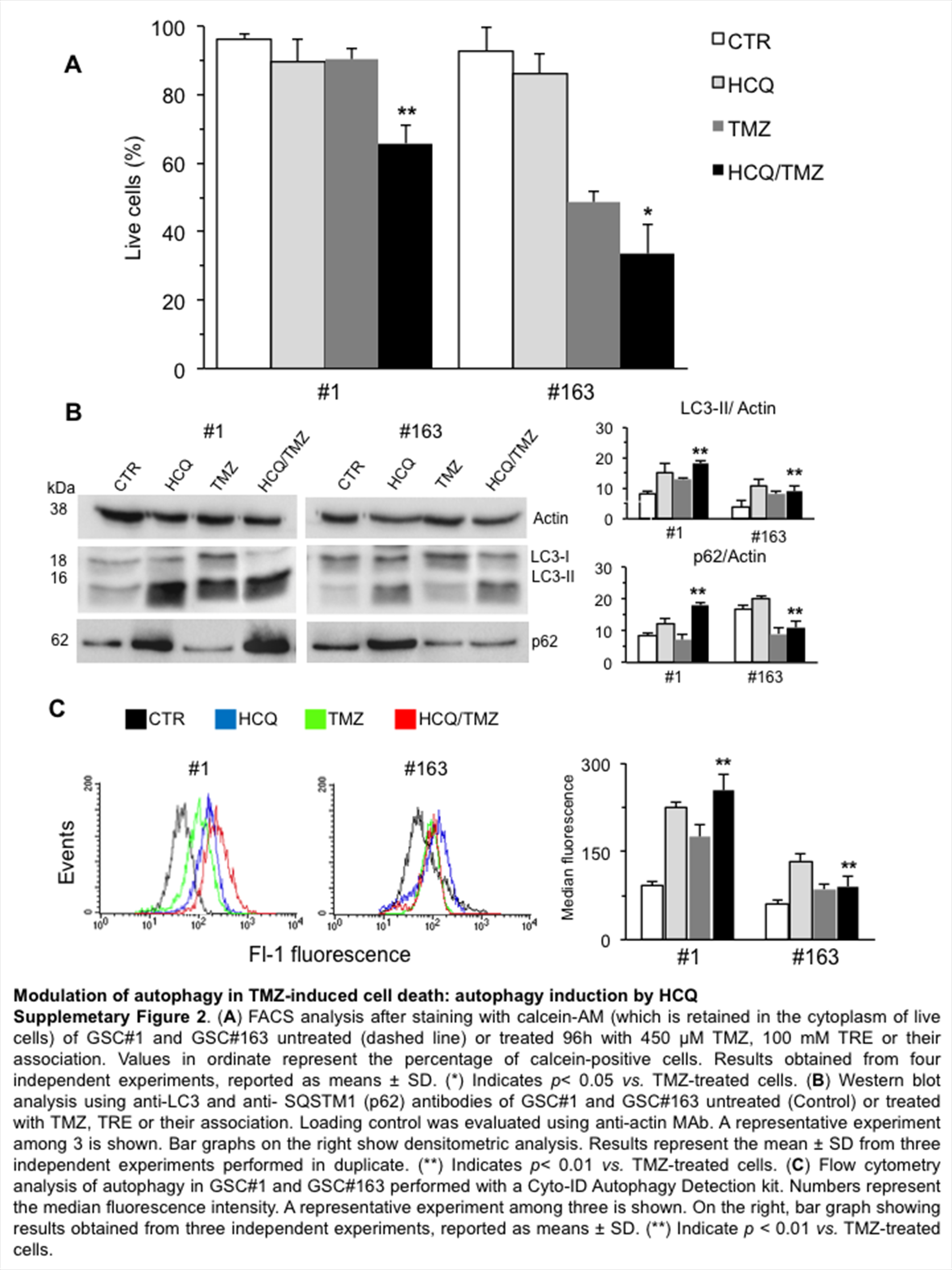

Supplement: Supplementary file 2 — Supplementary Figure 2 [file 41419_2018_864_MOESM2_ESM.tif]

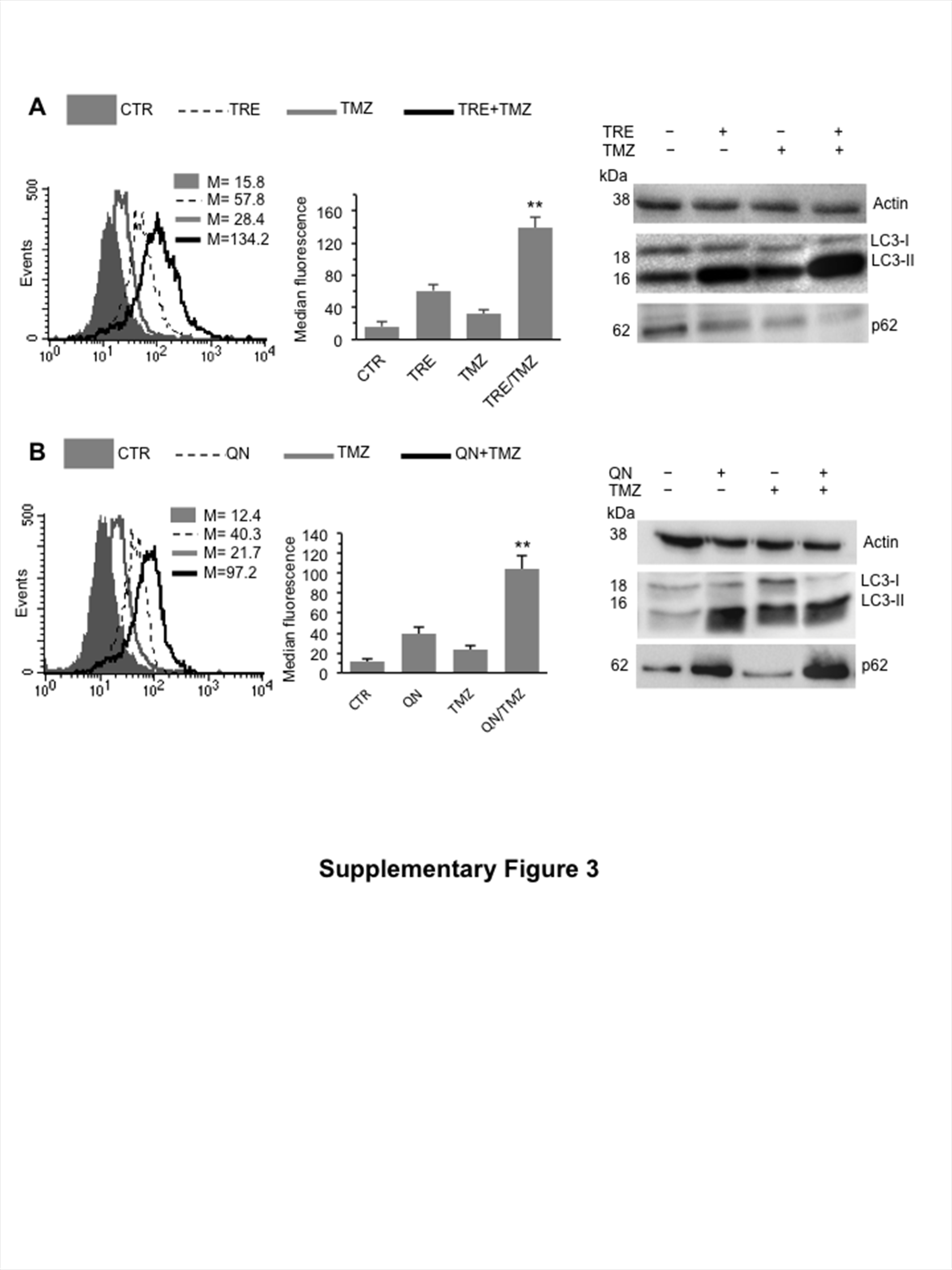

Supplement: Supplementary file 3 — Supplementary Figure 3 [file 41419_2018_864_MOESM3_ESM.tif]

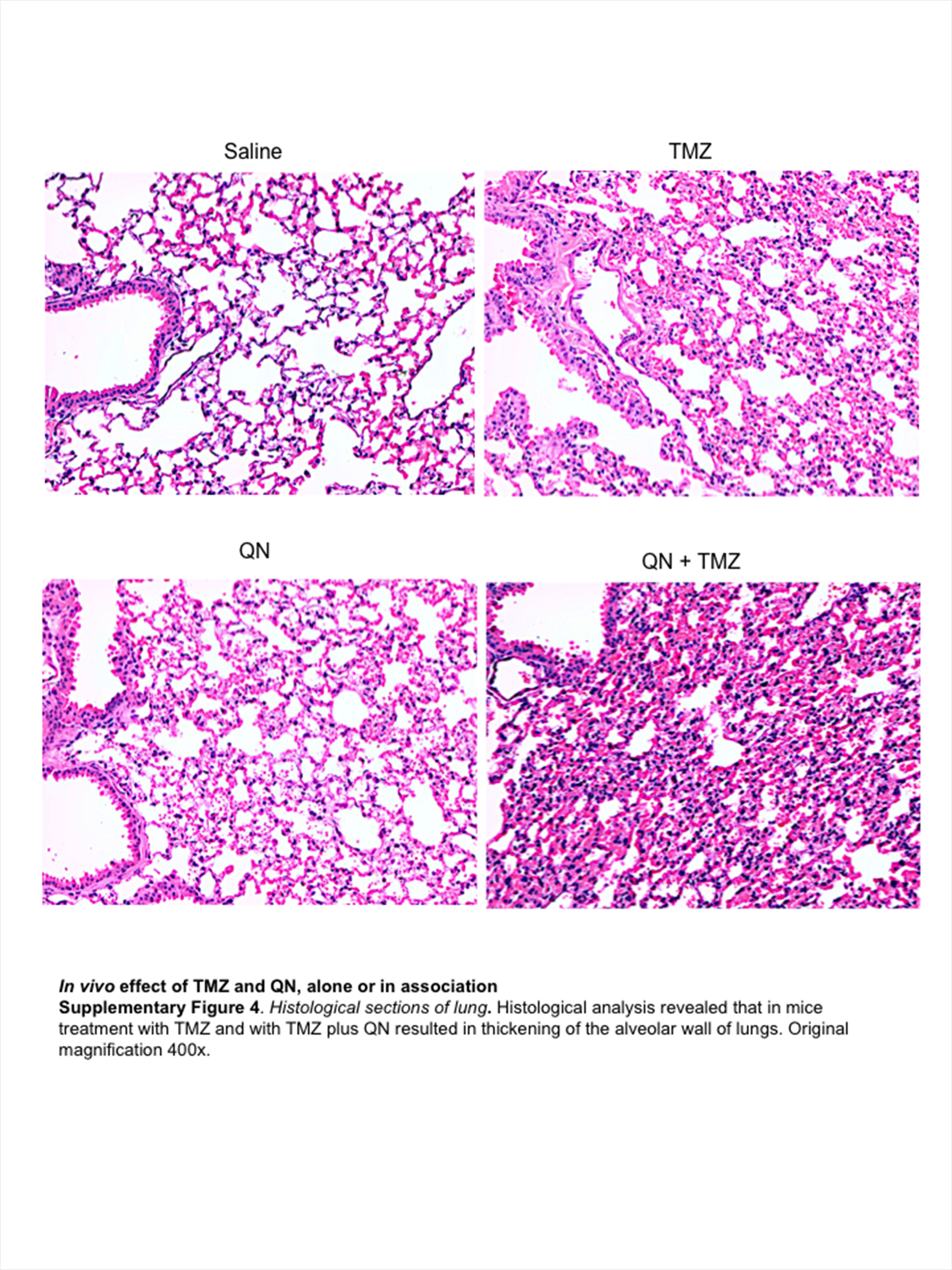

Supplement: Supplementary file 4 — Supplementary Figure 4 [file 41419_2018_864_MOESM4_ESM.tif]

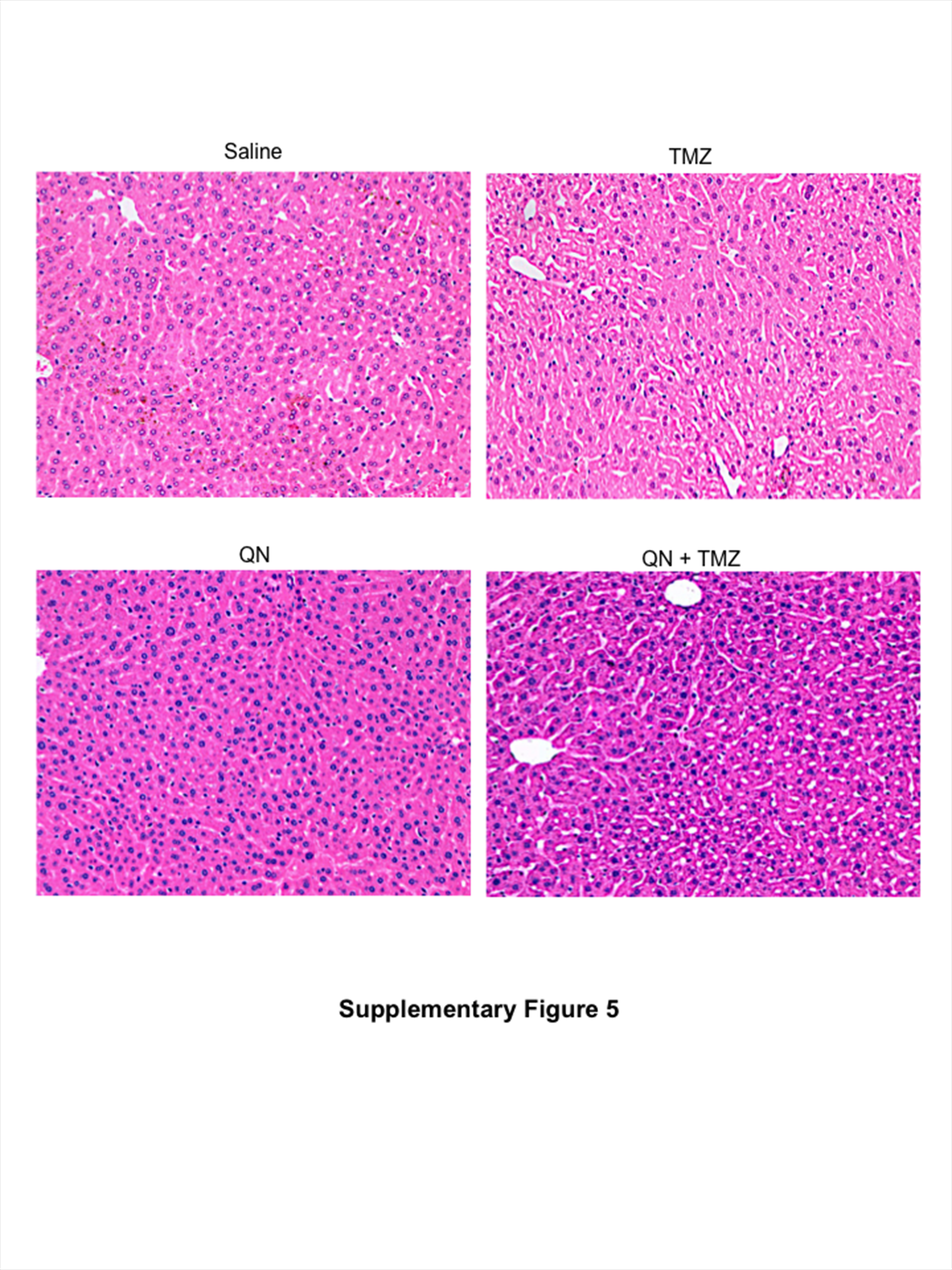

Supplement: Supplementary file 5 — Supplementary Figure 5 [file 41419_2018_864_MOESM5_ESM.tif]

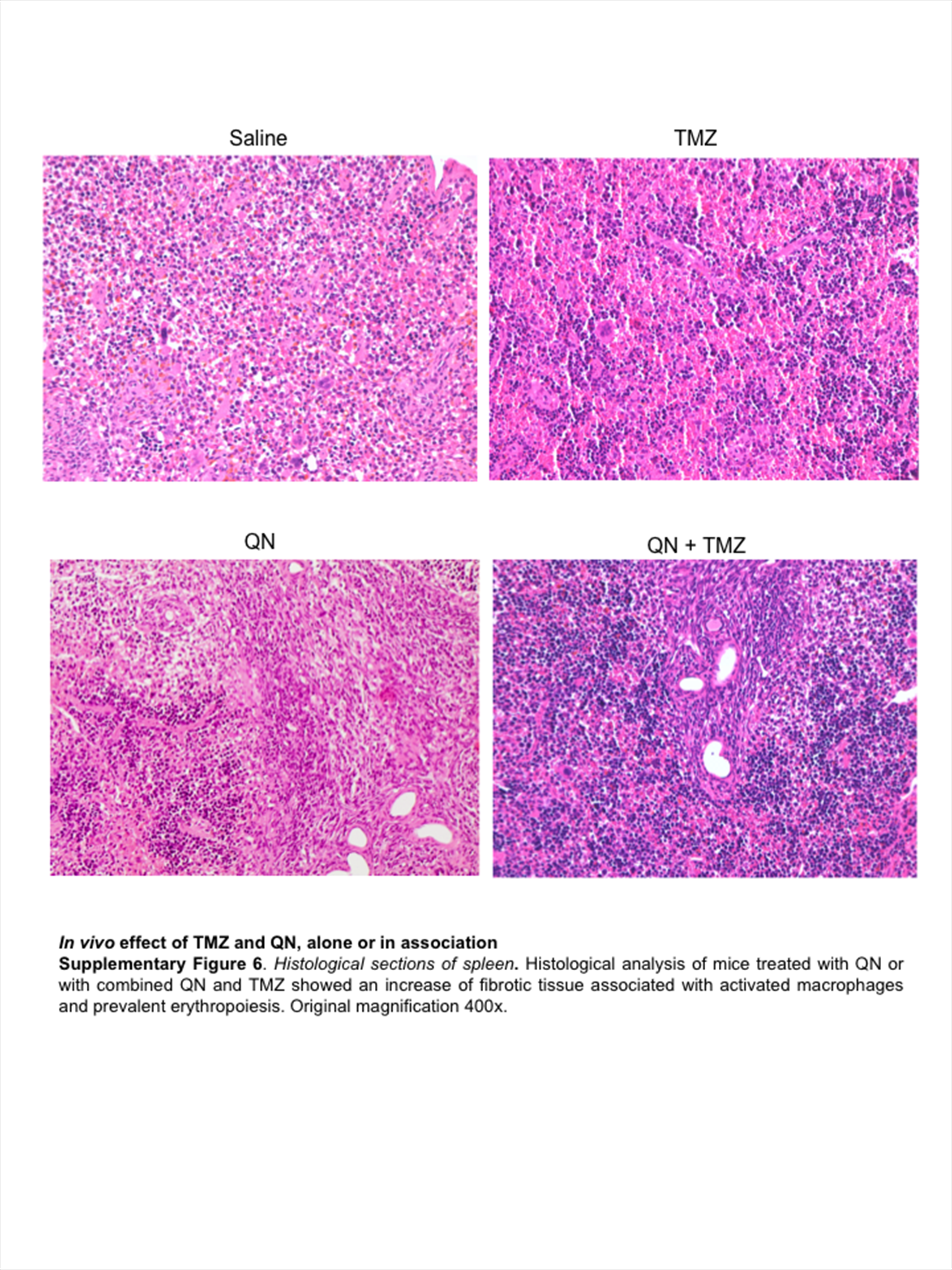

Supplement: Supplementary file 6 — Supplementary Figure 6 [file 41419_2018_864_MOESM6_ESM.tif]
